# Supplementary material for: Disinfection efficacy of sodium hypochlorite and glutaraldehyde and their effects on the dimensional stability and surface properties of dental impressions: a systematic review
Source: PeerJ. 2023 Feb 20;11:e14868. doi: 10.7717/peerj.14868 (PMC9948754; doi:10.7717/peerj.14868)
Supplement: Supplemental Information 1 [file peerj-11-14868-s001.docx]

| **Section and Topic** | **Item #** | **Checklist item** | **Location where item is reported** |
| --- | --- | --- | --- |
| **TITLE** | | |  |
| Title | 1 | The report is identified as a systematic review. | 1 |
| **ABSTRACT** | | |  |
| Abstract | 2 | The structured abstract includes Objective, Methods, Main results and Conclusions. | 2 |
| **INTRODUCTION** | | |  |
| Rationale | 3 | Described in the Introduction. | 4 |
| Objectives | 4 | Provided in the Introduction. | 4 |
| **METHODS** | | |  |
| Eligibility criteria | 5 | Stated in the Material and Methods. | 6 |
| Information sources | 6 | Stated in the Material and Methods. | 6 |
| Search strategy | 7 | Provided in the Material and Methods, as well as Table 1. | 6 |
| Selection process | 8 | Stated in the Material and Methods. | 7 |
| Data collection process | 9 | Stated in the Material and Methods. | 7 |
| Data items | 10a | Described in the Material and Methods. | 7 |
|  | 10b | Described in the Material and Methods. | 7 |
| Study risk of bias assessment | 11 | Stated in the Material and Methods. | 7 |
| Effect measures | 12 | This report is not a meta-analysis, and effect measures do not apply. | - |
| Synthesis methods | 13 | This report is not a meta-analysis, and synthesis methods do not apply. | - |
| Reporting bias assessment | 14 | Reporting bias assessment does not apply. | - |
| Certainty assessment | 15 | Certainty assessment does not apply. | - |
| **RESULTS** | | |  |
| Study selection | 16a | Described in the Results. | 8 |
|  | 16b | Described in the Results. | 8 |
| Study characteristics | 17 | Stated in the Results. | 8 |
| Risk of bias in studies | 18 | Described in the Results. | 9 |
| Results of individual studies | 19 | Provided in the Results, as well as Table 2 and 3. | 9 |
| Results of syntheses | 20 | This report is not a meta-analysis, and results of syntheses are not described. | - |
| Reporting biases | 21 | This report is not a meta-analysis, and reporting biases does not apply. | - |
| Certainty of evidence | 22 | This report is not a meta-analysis, and certainty of evidence are not described. | - |
| **DISCUSSION** | | |  |
| Discussion | 23a | Provided in the Discussions. | 17 |
|  | 23b | Discussed in the Discussions. | 17 |
|  | 23c | Discussed in the Discussions. | 24 |
|  | 23d | Discussed in the Discussions. | 24 |
| **OTHER INFORMATION** | | |  |
| Registration and protocol | 24a | Provided in the Material and Methods. | 6 |
|  | 24b | Indicated in the Material and Methods. | 6 |
|  | 24c | No amendments to information were made at registration or in the protocol. | - |
| Support | 25 | Described while submission. | - |
| Competing interests | 26 | Described while submission. | - |
| Availability of data, code and other materials | 27 | No other materials are publicly available. | - |

*From:*  Page MJ, McKenzie JE, Bossuyt PM, Boutron I, Hoffmann TC, Mulrow CD, et al. The PRISMA 2020 statement: an updated guideline for reporting systematic reviews. BMJ 2021;372:n71. doi: 10.1136/bmj.n71

For more information, visit: <http://www.prisma-statement.org/>
