# Supplementary material for: Disinfection efficacy of sodium hypochlorite and glutaraldehyde and their effects on the dimensional stability and surface properties of dental impressions: a systematic review
Source: PeerJ. 2023 Feb 20;11:e14868. doi: 10.7717/peerj.14868 (PMC9948754; doi:10.7717/peerj.14868)
Supplement: Supplemental Information 2 [file peerj-11-14868-s002.docx]

The rationale and the contribution of the current review:

A literature review by Kotsiomiti et al. (2008) preliminarily put forward the ideal disinfection methods according to different impressions materials. This review only focused on the dimensional stability of dental impressions, without investigating other surface properties of impressions which could be affected after disinfection. Other literature reviews by Chidambaranathan et al. (2017) and AlZain et al. (2019) comprehensively investigated the effect of physical and chemical disinfection methods on the surface properties of dental impressions. However, these studies neither investigated the effect of disinfectants on inhibiting bacterial growth or killing potential pathogenic bacteria, nor provided a concrete and feasible guideline of impression disinfection for clinical use. On the other hand, a systematic review and meta-analysis by Hardan et al. (2022) investigated the effect of several disinfectants on the microorganism colonization of dental impression materials. To the best of our knowledge, no systematic review has been conducted to evaluate the disinfection efficacy of frequently-used disinfectants and their effects on the dimensional stability and surface properties of dental impressions.

In this review, we systematically evaluate the disinfection efficacy of the two most frequently used disinfectants, sodium hypochlorite and glutaraldehyde, and their effects on the surface properties of four different dental impression materials. In addition, we focused on the several vital factors of disinfection procedures, including disinfection methods (immersion and spray), disinfection duration and concentrations of disinfectants. Based on the results of this review, we provided guidance for establishing standardized disinfection techniques of different impression materials.
